# Tumor in the veins: an abdominal perspective with an emphasis on CT and MR imaging

**DOI:** 10.1186/s13244-020-00854-x

**Published:** 2020-03-25

**Authors:** Ali Devrim Karaosmanoglu, Mehmet Ruhi Onur, Aycan Uysal, Deniz Akata, Mustafa Nasuh Ozmen, Musturay Karcaaltincaba

**Affiliations:** 1 Department of Radiology, Hacettepe University School of Medicine, 06100 Ankara, Turkey; 2 Department of Radiology, Gulhane Training and Research Hospital, 06010 Ankara, Turkey

**Keywords:** Abdomen, Vein, Tumor thrombus, Computed tomography, Magnetic resonance imaging

## Abstract

Endovenous tumor thrombus in abdomen should be accurately diagnosed as it is a significant finding that may change medical and surgical treatment approaches. As some underlying reasons for endovenous tumor thrombi are relatively rare and imaging findings may be quite subtle, they can be easily overlooked which may have important clinical consequences. In this paper, we described the various imaging aspects of endovenous tumor thrombi originating from various tumor types in different venous structures of the abdomen.

## Key points

- Venous involvement in HCC and RCC is a well-known entity; however, it should be kept in mind that various abdominal malignancies may present with tumoral thrombus in abdominal veins.
- Tumor thrombi in abdominal veins present as mildly enhancing and self-expanding filling defect in the venous structures.
- Familiarity with the imaging findings of tumoral thrombus in the abdominal veins may be important since management of patients including surgical approach may be changed.

## Introduction

Venous system of the abdomen is extremely complex with several interconnections between different vessels. Several systemic and local disorders may affect the abdominal veins, and early and correct detection is of crucial importance for treatment planning. Primary venous neoplasms as well as neoplasms of other adjacent anatomic structures may involve the abdominal veins during the course of the disease, and this involvement has important clinical and prognostic ramifications. In this article, we tried to present the imaging features of primary and secondary neoplastic involvement of the abdominal venous structures and highlight the important diagnostic clues for correct diagnosis.

## Classification of tumors in the abdominal veins

Tumors involving the abdominal veins may be primary or secondary from endoluminal extension from other organs (Table 1).

**Table 1:** Clinical and imaging features of tumoral thrombus

Vein	Diseases	Imaging feature
Portal vein	HCC Primary portal vein leiomyosarcoma (LMS)	Portal vein expansion with effacement and disruption of thin PV wall Heterogeneous contrast enhancement Diffusion restriction within the thrombus
Hepatic vein	HCC	Extension of tumoral tissue into the hepatic veins
Splenic vein	HCC PNET Gastric adenocarcinoma	Venous expansion with heterogeneous contrast enhancement mostly prominent in arterial phase
Inferior vena cava	Primary leiomyosarcoma of the IVC Adrenocortical carcinoma	The enlargement of IVC and contrast enhancement within the tumor thrombus
Renal vein	RCC Wilms’ tumor Renal AML Primary LMS	Contrast enhancing mass lesion extending from primary renal mass Enlargement of renal vein
Gonadal vein	RCC Gonadal vein LMS	Enlargement of gonadal vein with contrast enhancing filling defect
Pelvic veins	IVL Endometrial stromal sarcoma (ESS)	Uterine mass extending into the pelvic veins demonstrating heterogeneous enhancement
Inferior mesenteric vein	Colorectal cancer	Colorectal cancer extending into IMV
Great saphenous vein	LMS of the great saphenous vein	A heterogeneously enhancing mass lesion, expanding the vessel with intraabdominal extension

Primary venous tumors may originate from the vascular endothelium or smooth muscle cells within the vessel wall and may be benign or malignant. Leiomyosarcoma is a rare tumor however appears to be among the most common primary tumor of the abdominal veins [1]. Secondary tumor extension into the lumens of the abdominal veins appears to be more common compared to their primary counterparts. Tumors originating from the liver and the kidneys are well known for their propensity for venous invasion; however, several other tumors may also extend into the venous lumen [2].

## The role of CT and MRI in evaluating the abdominal veins

Computed tomography (CT) and magnetic resonance imaging (MRI) are the most commonly used imaging modalities for evaluating the abdominal veins. Due to its wide availability, CT is the most commonly used modality to evaluate the abdominal veins. Isotropic images reformatted in several planes from the source data allow accurate and thorough evaluation of the abdominal venous system. Proper protocoling is of fundamental importance for appropriate evaluation. Venous phase images allow demonstrating the endoluminal venous thrombus and its continuity with adjacent organs.

The filling defect is the main imaging finding. In secondary venous tumors, the endoluminal tumor thrombus typically demonstrates similar enhancement characteristics with the primary source. The thrombosed vessel typically appears enlarged, and perivenous tissues may also be infiltrated by the tumor thrombus. On the contrary, bland thrombi typically do not extend beyond the confines of the venous wall, which is an important finding for diagnosis. Typically, endoluminal thrombi from hepatocellular carcinoma (HCC), renal cell carcinomas (RCC), and neuroendocrine tumors avidly enhance in arterial phase whereas the other primary or secondary tumor thrombi are relatively hypoenhancing compared to these tumors. The enhancement pattern in tumor thrombi is typically heterogeneous in all phases [3].

MRI may also be used for assessment of the abdominal veins. MR angiography imaging with or without IV contrast use may be utilized for this purpose. T2-weighted (T2W) images in different planes are especially helpful for detecting endoluminal tumor thrombus. The presence of enhancement after IV contrast injection is helpful for differentiating tumor thrombus from bland thrombus. On T2W images, thrombus characteristically appear as heterogeneously hypointense whereas scattered hyperintense areas may be seen in precontrast T1-weighted (T1W) images due to blood products. Turbulent and pulsatile flow may mimic thrombus on time of flight images, and special attention should be paid for not to misdiagnose this pitfall as a real thrombus. Adequate timing is also important in interpreting MR images as mixture of nonopacified blood, and contrast agent may also mimic a thrombus [3–5].

Diffusion restriction is typical for tumor thrombus; however, bland thrombus may also appear as hyperintense on diffusion-weighted (DW) images. Therefore, postcontrast T1W images are critical for differentiating tumor thrombus from its bland counterpart.

### Portal vein

The portal vein (PV) is the main inflow vessel of the liver, supplying around 80% of the blood inflow into the liver. It typically measures 12.6 ± 1.7 mm in diameter with a length of around 8 cm [6]. It is formed by junction of the splenic vein and the superior mesenteric vein behind the pancreatic head at the level of L2 vertebral body. The vessel wall is thin with no valves that prevent backflow. Several neoplastic diseases may affect the PV, and early detection of involvement of PV is of critical importance for treatment planning and prognostic assessment.

**Fig. 1:**
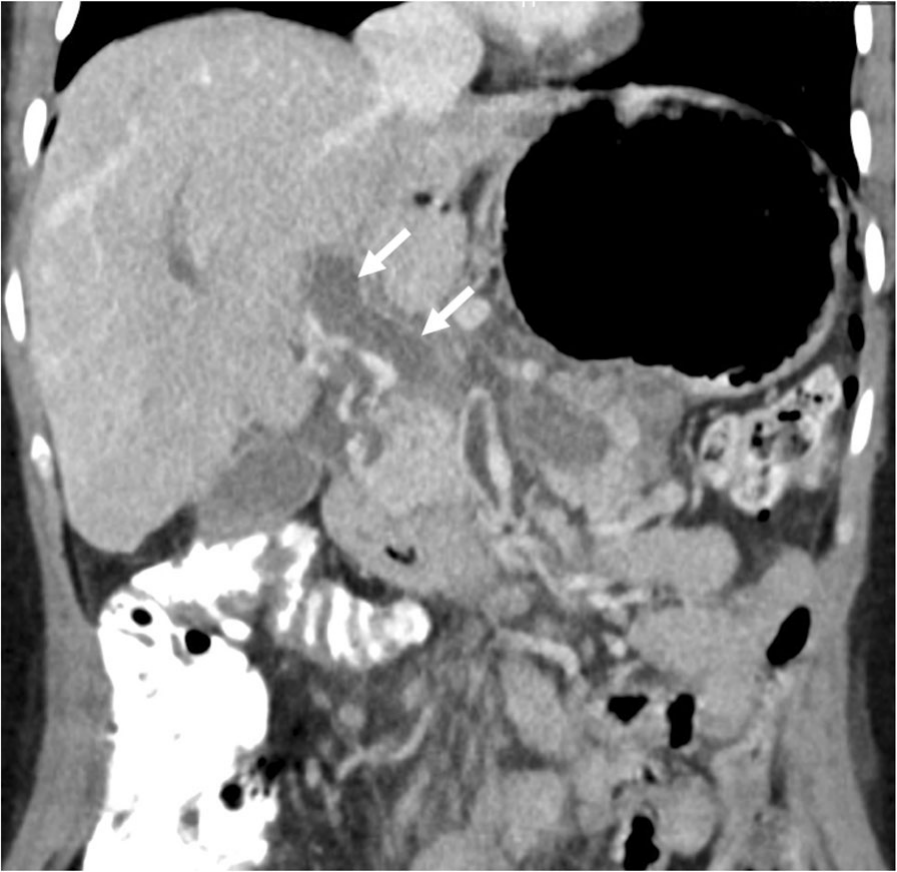
A 34-year-old female with myelofibrosis underwent splenectomy 3 days ago now presenting with right upper quadrant pain and elevated liver enzymes. Coronally reformatted postcontrast CT image demonstrates low attenuating filling defect within the main portal vein (arrows) with no enhancement consistent with bland thrombus

**Fig. 2:**
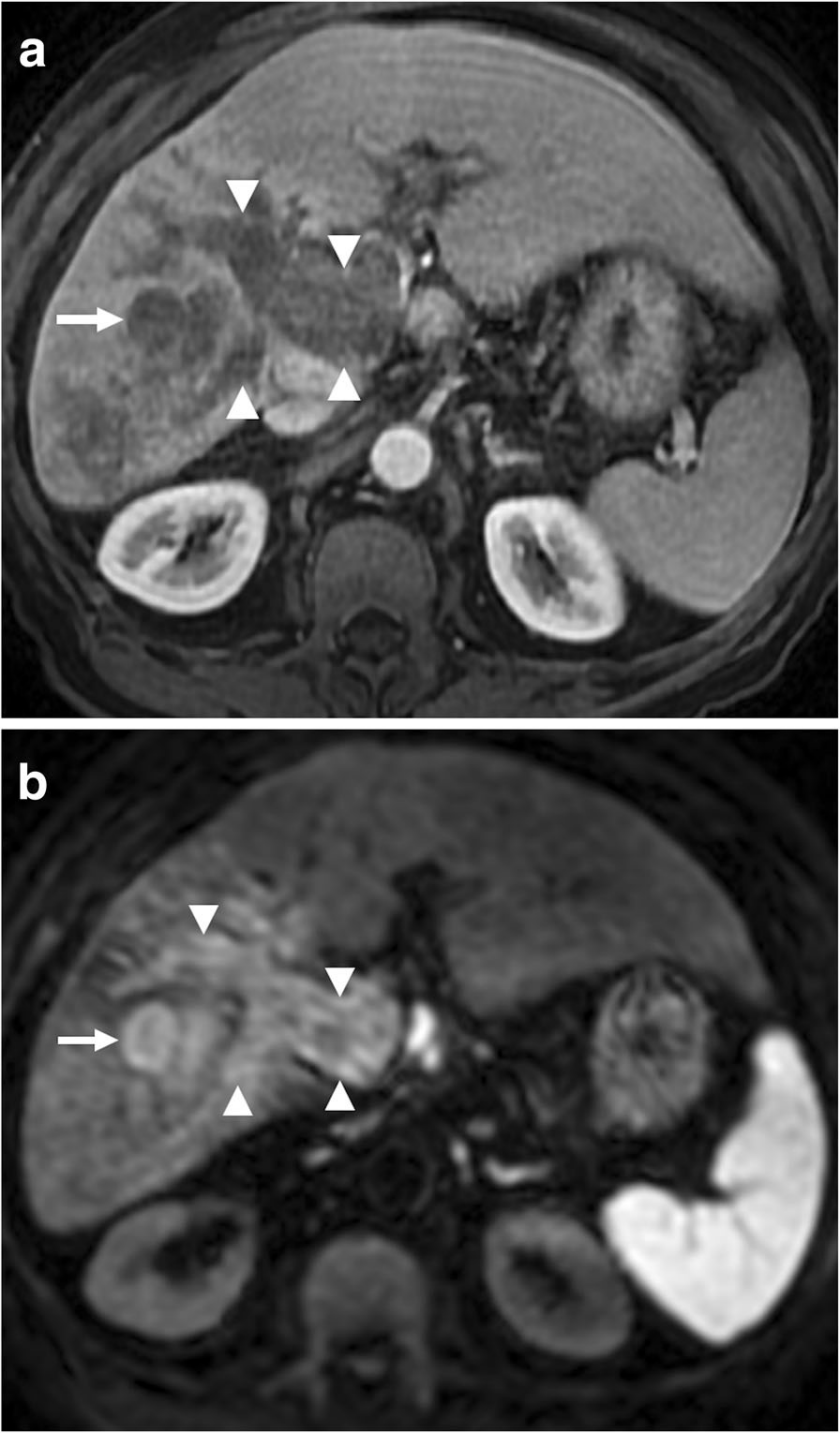
A 76-year-old male with known cirrhosis presents with elevated liver enzymes and alpha fetoprotein (AFP) levels. **a** Axial postcontrast T1W MR image demonstrates the HCC focus within the right liver lobe (arrow) with associating tumor of the main and right portal veins (arrowhead). Note contrast enhancement within the thrombus and its expansile nature extending outside the confines of the portal vein. **b** DWI sequence shows high signal intensity representing diffusion restriction of both the main tumor (arrow) and its intravenous component (arrowhead)

Hepatocellular carcinoma (HCC) is the most common tumor that invades the PV. Macrovascular invasion is a poor prognostic sign and an important contraindication for liver transplant. Five-year survival after surgical resection in these patients is poor around 12–39%, in contrast to 59% in patients without. Palliative techniques are mostly adopted in these patients over potentially curative interventions [7]. Therefore, it is of crucial importance to detect tumor thrombus within the PV lumen [8–10]. As bland thrombus may also be seen within the PV lumen in patients with cirrhosis, differentiation of these two different clinical entities should be done before any treatment planning. Malignant invasion of the PV by HCC is not rare and may be observed in 12–30% of these patients [11]. Imaging is almost always the only approach for non-invasive diagnosis of tumor thrombus within the PV [12]. Percutaneous biopsy may give erroneous results when adjacent HCC mass is biopsied instead of the luminal thrombus, and background cirrhotic liver may pose increased risk for bleeding during and after percutaneous biopsy [7]. CT and MRI are both commonly used for detection of PV invasion with a high sensitivity and specificity. Several imaging features may be helpful for detecting luminal tumor thrombus. The expansile nature of the thrombus with effacement and disruption of thin PV wall is an important imaging feature that favors tumor thrombus over bland thrombus (Figs. 1 and 2). The contrast enhancement within the thrombus following the contrast uptake pattern of the main tumor mass within the liver parenchyma is the most specific sign for tumor thrombus. Diffusion-weighted imaging (DWI) was recently proposed as a potentially important tool for differentiating bland thrombus from its neoplastic counterpart with variable reported success rates [7, 12]. Both bland thrombus and tumor thrombus may show diffusion restriction, and differentiation may be problematic in some patients [12].

**Fig. 3:**
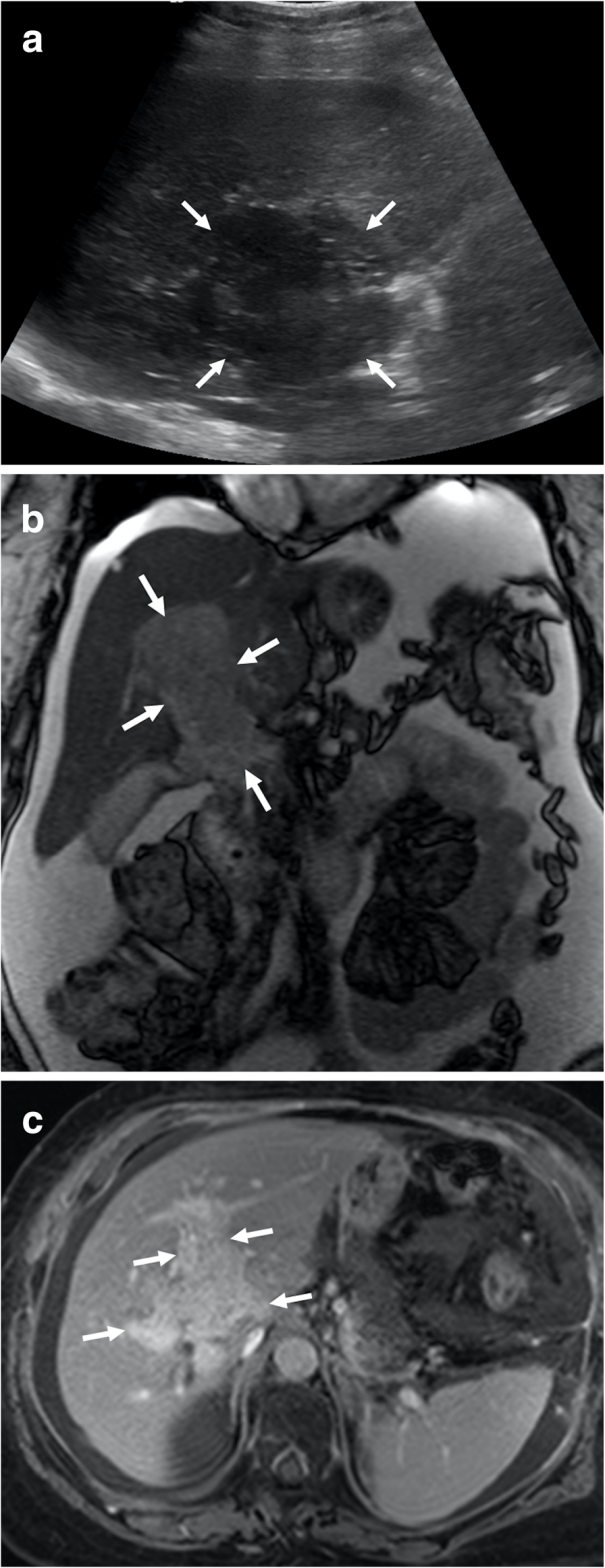
A 62-year-old female with unremarkable medical history now presents with right upper quadrant pain and weight loss. **a** Abdominal US study shows a large, hypoechoic solid mass with ill-defined borders (arrows) at the liver hilum. **b** Coronal T2W MR image demonstrates an infiltrative, expansile mass within the main portal vein and its distal branches (arrows). **c** Axial plane postcontrast equilibrium phase T1W MR image demonstrates intense contrast enhancement of the mass (arrows) suggestive for a tumor thrombus over a bland thrombus. There were no associating imaging findings suggestive for parenchymal chronic liver disease or parenchymal HCC focus. Percutaneous image guided biopsy revealed portal vein leiomyosarcoma

**Fig. 4:**
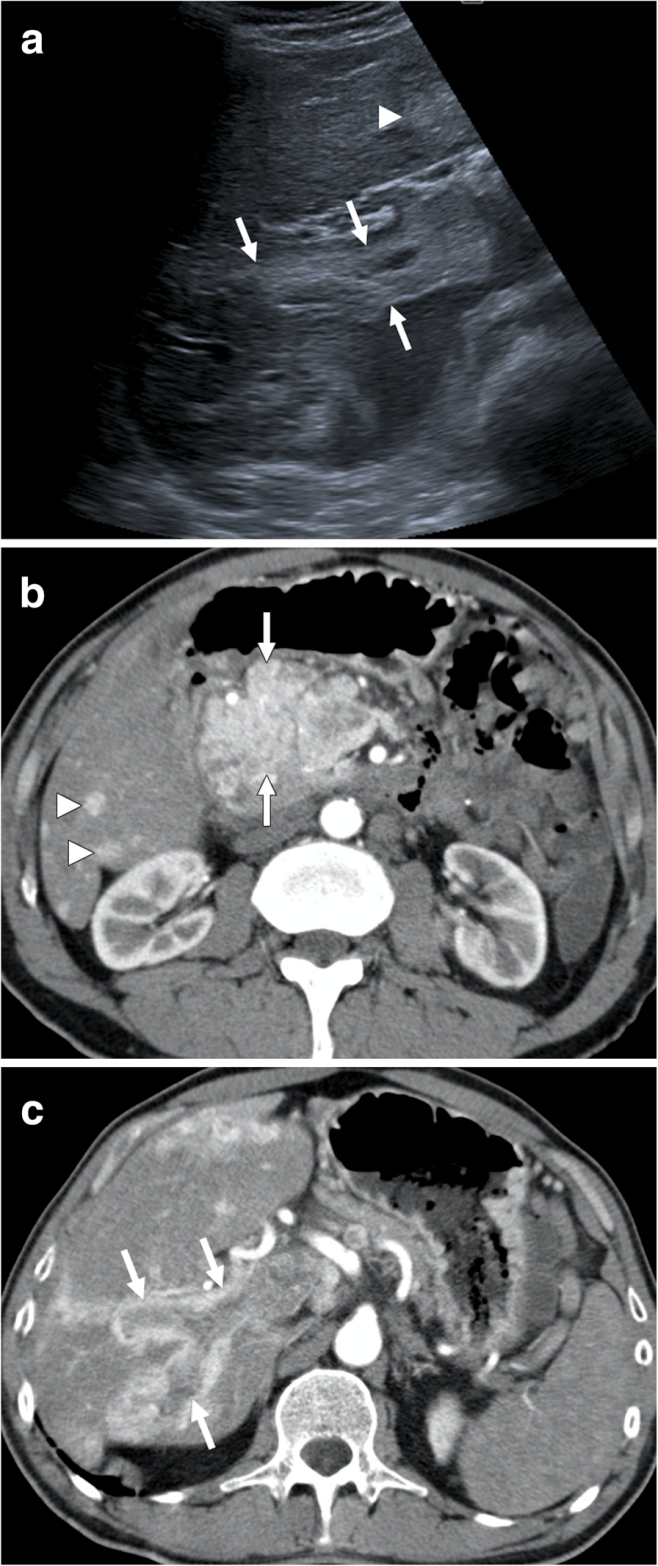
A 55-year-old male with no past medical history presented with epigastric pain and severe weight loss. **a** Gray-scale ultrasound image demonstrated a large mass in the epigastric area (not shown) with hyperechoic thrombus within the main portal vein (arrows). There was also a focal solid lesion within the left liver lobe (arrowhead) suggestive for metastatic disease. **b** Axial plane postcontrast arterial phase CT image shows avidly enhancing mass (arrows) located within the pancreatic head. Imaging findings were highly suggestive for pancreatic neuroendocrine tumor (NET). Also, note is made of small enhancing nodules within the liver parenchyma suggestive for metastases (arrowheads). **c** Axial plane postcontrast arterial phase CT image shows enlarged right portal vein with its lumen filled with contrast enhancing tumor thrombus (arrows). Percutaneous image-guided biopsy from the mass revealed pancreatic NET

Portal vein leiomyosarcoma is an extremely rare clinical entity and, to the best of our knowledge, only around 5 patients ever reported [13]. Despite the rarity of the disease, there is not enough data in the literature about its demographics, but it appears like it is mostly a disease of female population [13]. Surgical resection appears to be the only potentially curative treatment approach. This extremely rare tumor may easily be confused with more common causes of tumor thrombus in PV, such as HCC. However, the absence of chronic liver disease and the primary tumor within the liver parenchyma are the key imaging features for the correct diagnosis. As the tumor significantly expands the PV with associating heterogeneous enhancement, the tumor thrombus may be easily favored over its bland counterpart (Fig. 3). Tumor thrombus due to pancreatic neuroendocrine tumors (PNET) should be considered in the differential diagnosis, as these tumors may also cause extensive thrombosis in the splenoportomesenteric venous complex (Fig. 4).

### Hepatic veins

**Fig. 5:**
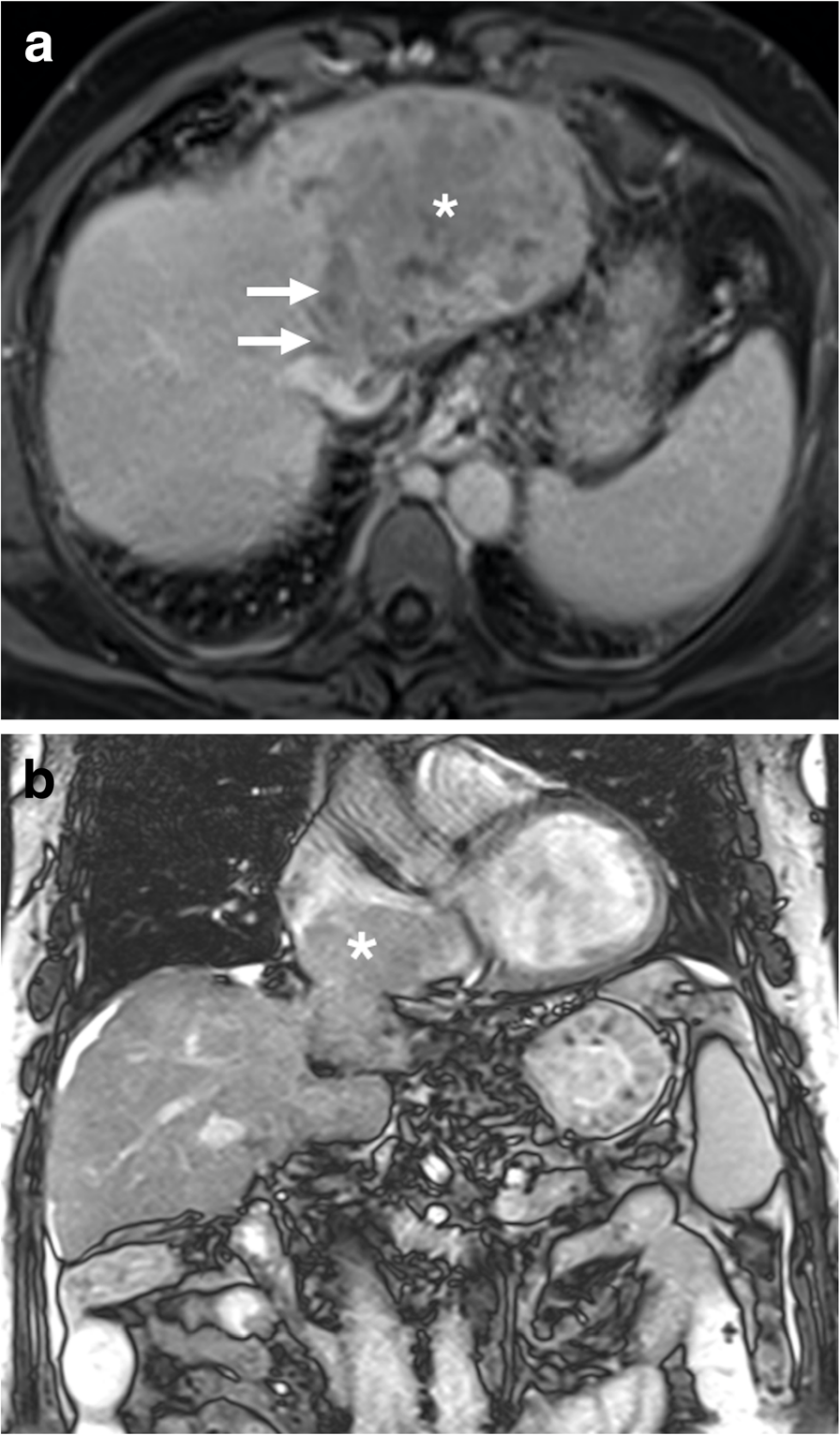
A 60-year-old male with known hepatitis B-related chronic parenchymal liver disease now presents with abdominal pain, low extremity edema, elevated liver enzymes, and elevated AFP levels. **a** Axial plane postcontrast T1W MR image demonstrates a heterogeneously enhancing solid mass within the left liver lobe (asterisk) with extending tumor thrombus to the left hepatic vein (arrows) and IVC. **b** Coronal T2W MR image of the same patient demonstrates the extension of the tumor thrombus in the right atrium (asterisk)

The hepatic veins form the most important outflow tract which eventually drain into the right atrium. Hepatic vein invasion by HCC, unrelated to the size, is a grave prognostic indicator with almost no hope for cure [14, 15]. Therefore, correct identification of this finding is critical for proper patient triaging and treatment. Despite the fact that this finding is generally regarded as a contraindication for surgery, there are some reports suggesting benefit for surgery in select groups [16]. The tumor thrombus may extend into the heart in advanced stage patients. Both CT and MRI may be used with superior precision to detect and evaluate the extent of hepatic vein tumor thrombus (Fig. 5). Sagittal and coronal reformatted images may also provide incremental value for evaluation.

### Splenic vein

Endovenous tumor thrombus in the splenic vein is relatively rare as compared to the portal vein. Pancreatic adenocarcinomas frequently invade this vessel, but endovenous tumor thrombus is rare. HCC is the other tumor that needs to be mentioned for tumor thrombus within the splenic vein lumen [17]. PNETs are rare tumors with an incidence of lower than 1 per 100,000 persons per year [18, 19]. Splenic vein tumor thrombus in these patients has rarely been addressed in the literature, but the incidence may be higher than expected [18]. Splenic vein tumor thrombus appears to be more common in patients with non-functional PNETs when compared to their functional counterparts. The early detection of venous tumor thrombus is important as the surgical planning may differ significantly based on the presence of this finding. Despite the mentioned importance of this finding, they are underreported in imaging studies [18].

**Fig. 6:**
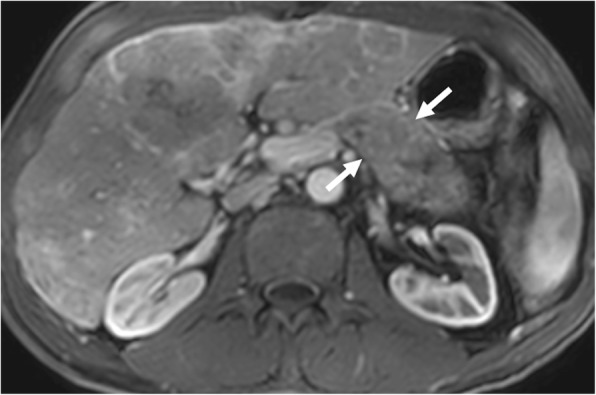
A 60-year-old male with known metastatic gastric NET. Axial plane portal phase postcontrast T1W MR image shows extensive tumor thrombus within the splenoportomesenteric venous junction extending into the splenic vein (arrows). Note that the endovenous tumor infiltrates beyond the confines of the splenic vein

On cross-sectional imaging, these tumor thrombi appear to follow the expected contrast enhancement pattern of the primary PNETs. Arterial phase hyperenhancement with relatively less enhancement of portal venous phase is a common finding. The tumor thrombus typically expands the vessels and propagates along the vessel trajectory (Fig. 6). The extension to the portal vein is a contraindication for distal pancreatectomy and, therefore, should be mentioned in the final report [18].

**Fig. 7:**
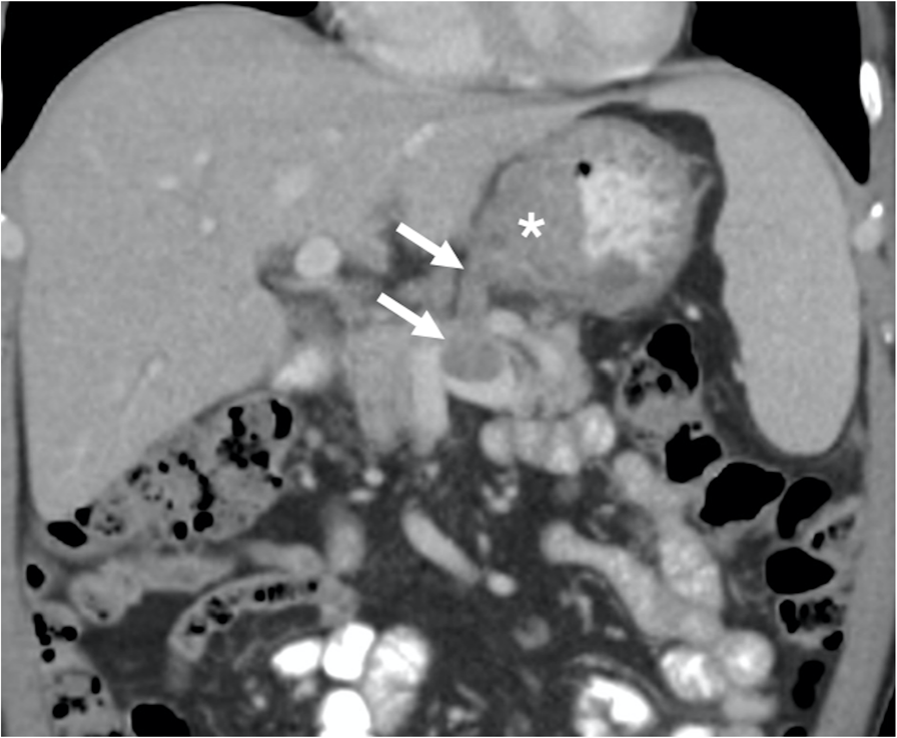
A 45-year-old male with newly diagnosed gastric cancer. Coronally reformatted postcontrast CT image shows the primary tumor in the gastric wall (asterisk) with tumor thrombus (arrows) extending into the splenic vein

Tumor thrombus within the splenoportomesenteric venous axis due to gastric adenocarcinoma is rare with a reported incidence of 1.2% [20]. The early detection is critical as it may change the surgical treatment planning in these patients (Fig. 7). Hypercoagulability and decrease in portal venous flow in patients with metastatic lymph nodes around the portal vein have been proposed as potential explanations for this rare occurrence [21].

### Inferior vena cava

The inferior vena cava (IVC) is the largest vein in the body and basically drains the lower extremities and the genitourinary system. Imaging plays a key role for detection of primary and secondary neoplastic diseases of the IVC. Both CT and MRI provide excellent images of IVC, in several different planes, in a short period of time. The IVC is typically better visualized in the portal venous phase (60–70 s after injection of the IV contrast agent). Several different primary and secondary neoplastic diseases may affect the IVC, and proper imaging is crucial for proper planning.

Primary leiomyosarcoma of the IVC is a rare malignant tumor that originates from the smooth muscle cells located in the vessel wall. It accounts for ~ 0.5% of adult soft tissue sarcomas, affecting 1/100,000 of all adult malignancies [22]. The overall 10-year survival was reported to be around 14.2% [23]. In patients who are not amenable to surgery, the estimated survival time is extremely poor as around 1 month and around 34 months who are candidates for surgery [24].

**Fig. 8:**
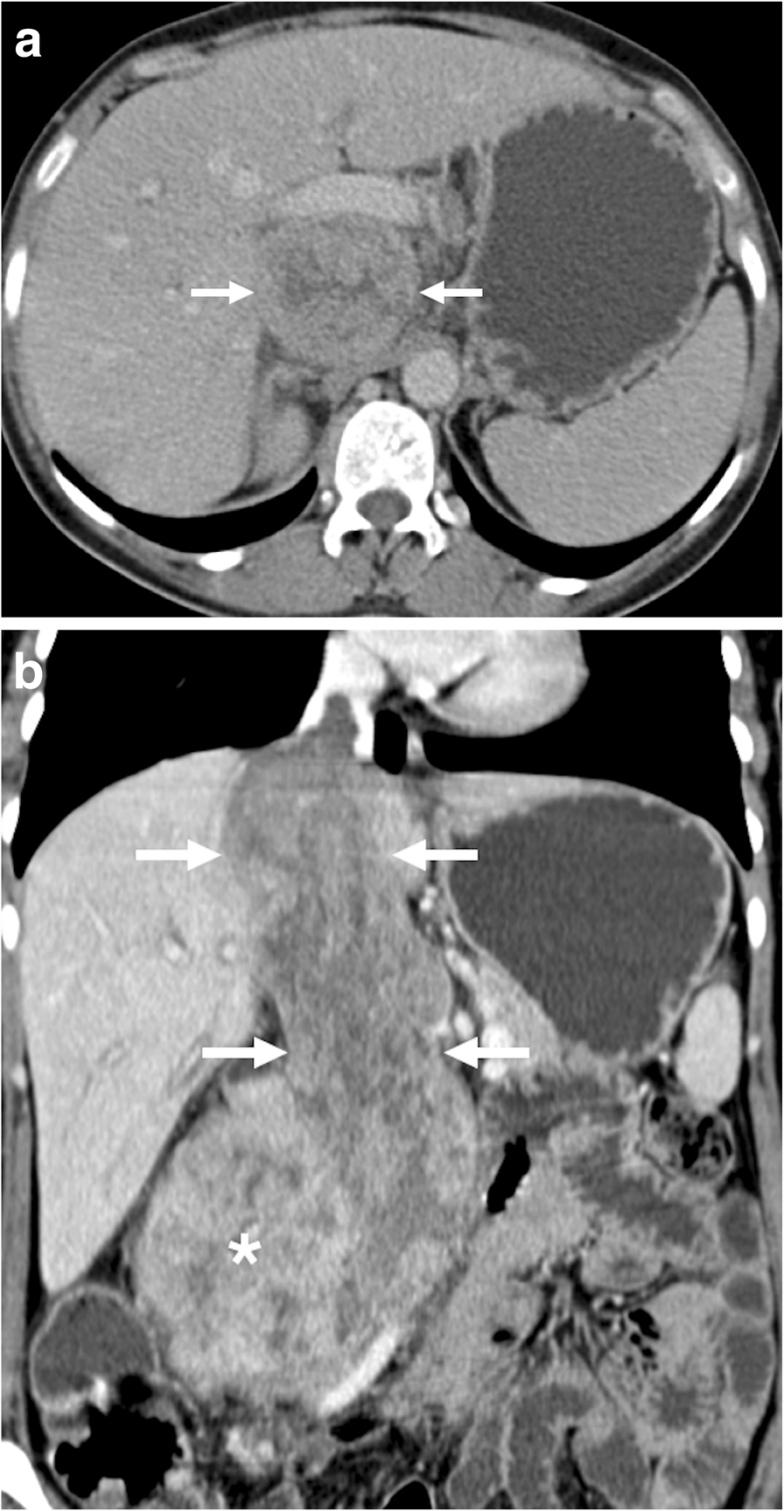
A 35-year-old female with no significant past medical history presented with palpable mass in the abdomen and bilateral leg swelling. **a** Axial plane postcontrast CT image shows a contrast enhancing expansile mass within the IVC lumen (arrows). **b** Coronally reformatted postcontrast CT image shows the extension of the tumor along the trajectory of the IVC (arrows) with prominent extraluminal extension (asterisk). Biopsy confirmed leiomyosarcoma of the IVC

The exact location of the tumor is critical both for surgical planning as well as for prognostic stratification. The tumor arising from the lower third of the IVC (caudal to the origins of the renal veins) has the worst prognosis (practically 0.0% 10-year survival rate), whereas the tumors arising from the middle segment (between the hepatic veins and the renal veins) have the best prognosis (around 34.4% 10-year survival rate) [25, 26]. Recurrences are common, and the recurrent tumor may be biologically more aggressive than the original tumor itself [27]. The tumor may grow predominantly within the lumen or outside the lumen. The form that grows toward the pericaval soft tissue planes is the more common type (around 73%). This type may be different to differentiate from other retroperitoneal malignancies. The predominantly intraluminal growing variant of the IVC leiomyosarcoma in hepatic segment may sometimes be difficult to differentiate from HCC and may present with symptoms of Budd-Chiari syndrome due to hepatic venous outflow obstruction [23]. The tumor mostly appears as a large mass (typically greater than 10 cm in diameter) with lobulated borders. The contrast enhancement is mostly heterogeneous with possible internal necrotic areas and blood elements. Predominantly cystic mass and intratumoral calcifications are unusual [27, 28] (Fig. 8).

**Fig. 9:**
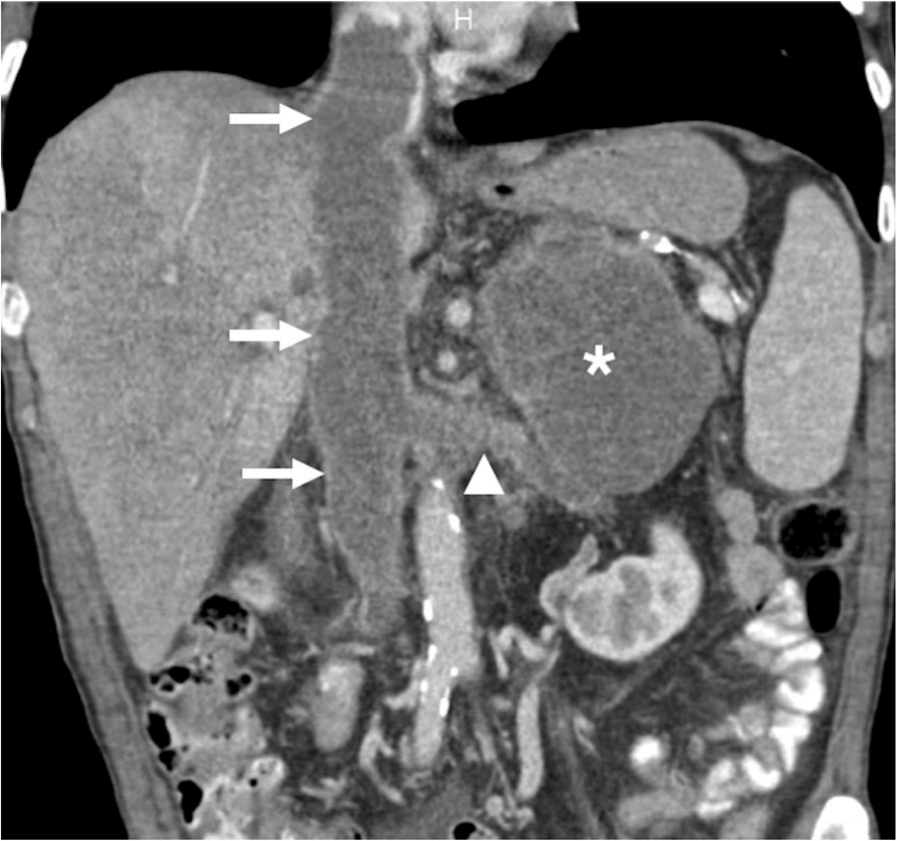
A 60-year-old male with no past medical history presented with left flank pain and weight loss. Coronally reformatted postcontrast CT image shows a left adrenal mass (asterisk) invading the left renal vein (arrowhead) and IVC (arrows). Surgical resection revealed adrenocortical carcinoma with tumor thrombus extending into the left renal vein and IVC

Adrenocortical carcinoma (ACC) is a rare malignancy with an incidence of 0.7–2.0 cases/million. This tumor may be detected at any age, but its distribution is mostly bimodal with the first peak in the first decade and the second peak in the fourth to fifth decades [29]. Women are slightly more frequently affected (55–60%) than men [23]. The tumors are most commonly detected at an advanced stage and are of large size (mostly greater than 6 cm in diameter) upon diagnosis [30]. Vascular or visceral organ involvement is not rare. Calcifications may be seen within the tumor tissue. As the tumor is mostly of large size at the time of clinical presentation, other organs tumors originating from the liver or the right kidney may be initially considered. In these patients, reformatted images at different planes may be helpful for differential diagnosis. Involvement of IVC in patients with ACC is rare, but it poses real challenge for surgical resection, which is currently the only potentially therapeutic approach for these patients [31]. Reformatted images may also be helpful for diagnosing renal vein and IVC involvement. The enlargement of these vessels beyond the confines of the vessel wall and contrast enhancement within the tumor are the two most reliable imaging features for correct diagnosis (Fig. 9).

**Fig. 10:**
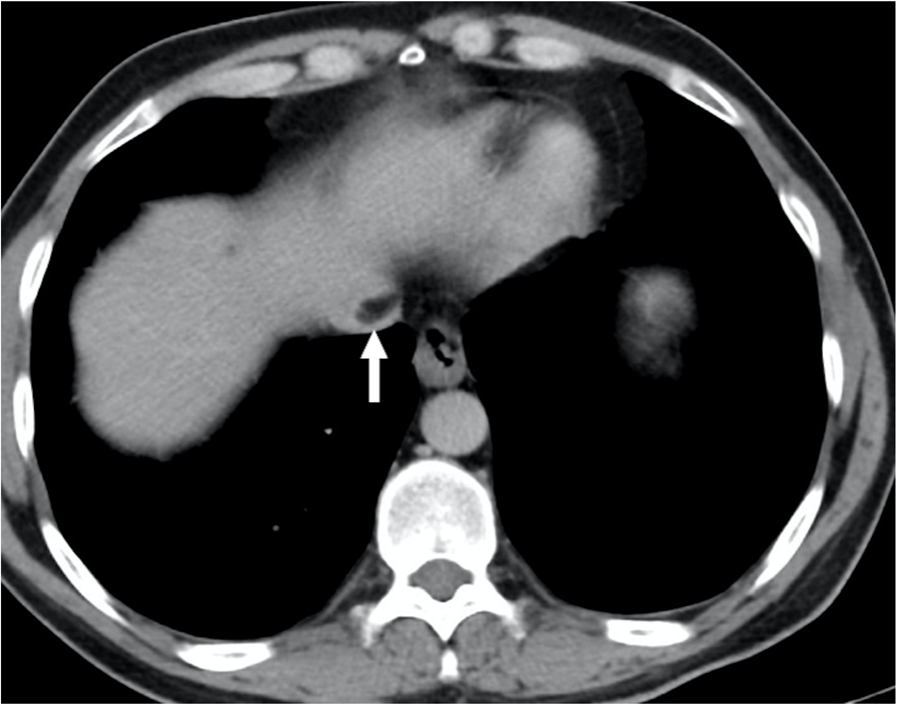
A 40-year-old male with no past medical history underwent abdominal CT examination for nonspecific abdominal pain. Axial plane postcontrast CT image demonstrates protrusion of pericaval fat into the IVC lumen (arrow) mimicking an endoluminal thrombus

Several imaging pitfalls that may simulate a true thrombus in the IVC lumen should be recognized promptly by the imaging specialists to prevent unnecessary test and treatment. Pseudolipoma of the IVC refers to adipose tissue in the pericaval area that surrounds the IVC mimicking an intracaval fat-containing lesion. It is reported to be present in CT exams of the 0.5% of the adult population [32]. It is typically seen in medial and posterior aspects of the IVC lumen at or superior to the confluence of the hepatic veins and IVC [33, 34] (Fig. 10). This paracaval fat may be seen in different configurations on serial CT exams due to differences in respiratory depth or pressure [32]. Reformatted images are helpful for differentiating these pseudolesions from true fat-containing endoluminal caval tumors. This pseudolesion may appear more prominent in patients with cirrhosis due to shrunken right liver lobe tilts the IVC further and may accentuate the pericaval adipose tissue.

Flow-related pseudothrombus appearance in IVC may be seen in some cross-sectional imaging studies, most commonly at the level of the renal veins. This finding is related to the mixture of enhanced venous blood returning from the renal veins and non-enhancing blood ascending from the lower extremities [35]. The awareness of this imaging phenomenon is the key to prevent misdiagnosis, and reformatted images may also be helpful. In patients with IVC filter, signal loss due to metallic content of the filter may also give rise to pseudothrombus phenomenon in the IVC. The typical location of the filter below the IVC and detailed patient history are fundamental for avoiding this pitfall.

### Renal veins

**Fig. 11:**
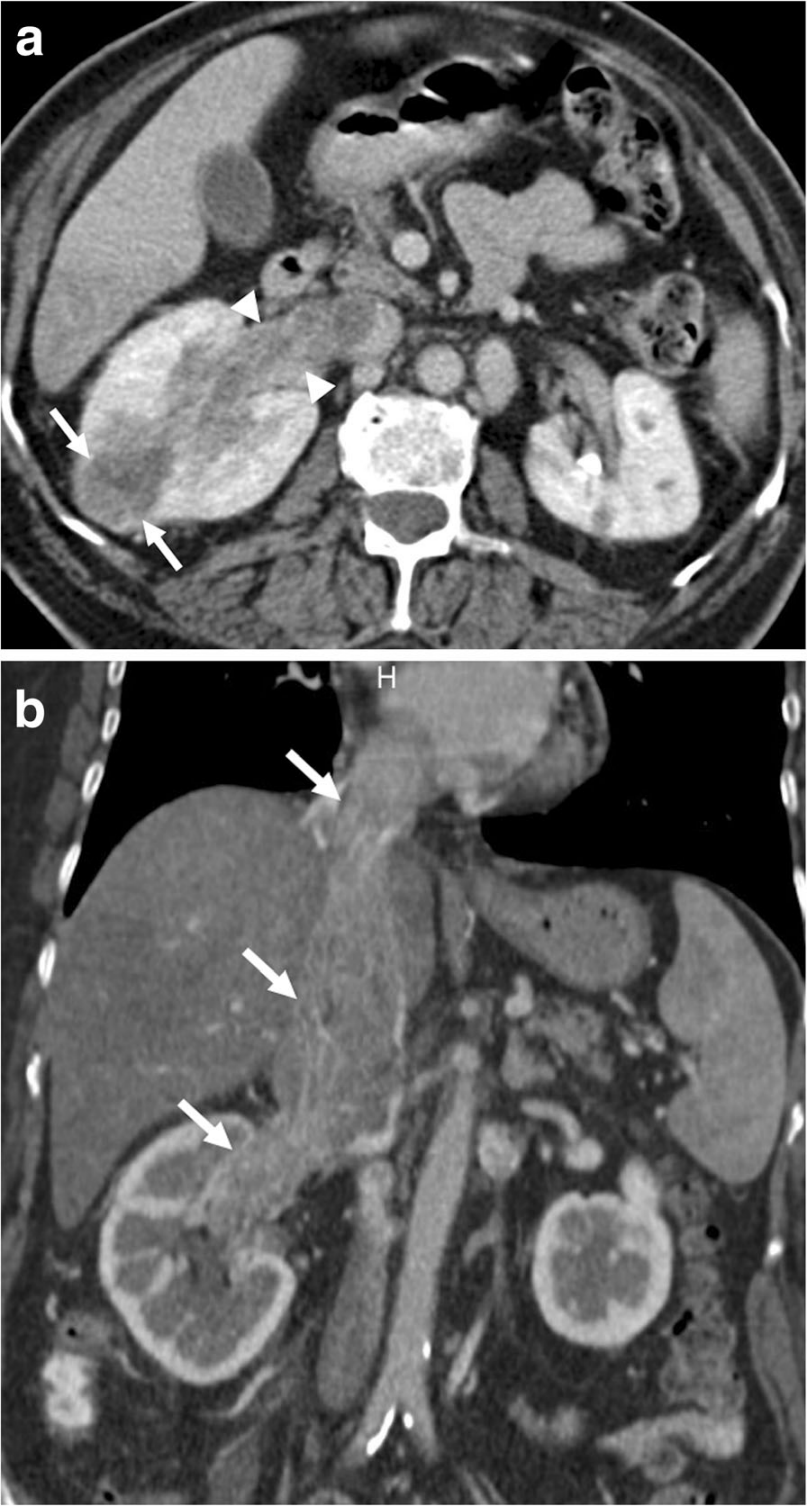
A 66-year-old female with no significant past medical history presented with right flank pain, hematuria, and lower extremity edema. **a** Axial plane postcontrast CT image shows peripherally located, ill-defined, heterogeneously enhancing solid mass (arrows) within the right kidney parenchyma suggestive for RCC. There was tumor extension into the right renal vein (arrowheads). **b** Coronally reformatted postcontrast CT image demonstrates infiltrative, expansile, heterogeneously enhancing tumor thrombus extending from the right renal vein to the IVC and the right atrium (arrows)

The renal veins may be commonly affected with tumor thrombus in patients with renal cell carcinoma (RCC). Besides RCC, angiomyolipomas and rare primary renal vein malignancies may affect these vessels by forming tumor thrombi. Imaging plays a critical role in the diagnosis, treatment, and follow-up of these patients. One of the peculiar features of RCCs is their tendency to invade the venous system with extensions into the renal vein and the IVC, and this involvement was reported to be observed in 23% and 7% of the cases, respectively [36]. Both CT and MRI have high accuracy for detecting tumor thrombus, assessing its extension, and differentiating it from bland thrombus [2]. Despite its relatively common occurrence, the presence of renal vein wall invasion and extension into the perivascular soft tissue planes are rare [37, 38]. The presence of enhancement, reflecting the enhancement pattern of the main tumor mass, is the most beneficial imaging clue for diagnosing tumor thrombus over a bland thrombus. The presence of vascular expansion and avid enhancement on fluorodeoxyglucose on positron emission tomography (18F-FDG-PET) are other helpful features [39]. The extension of the tumor thrombus into the IVC may make surgical treatment approaches even more challenging. Level of the extension is important for treatment planning, and the extent was stratified according to Mayo system [40]:

- Level 1: Refers to the thrombi that extend into IVC no more than 2 cm above the renal vein.
- Level 2: Refers to the thrombi extending into IVC to more than 2 cm above the renal vein but not to the hepatic vein.
- Level 3: Refers to thrombi extending above the hepatic veins but below the diaphragm.
- Level 4: Refers to IVC thrombi extending above the diaphragm or into the right atrium (Fig. 11).

Levels 1 and 2 thrombi represent 40% of IVC tumor thrombi while level 3 accounts for 40% and, finally, level 4 makes up the remaining 10% [41].

Wilms tumor is the most common primary renal cancer in pediatric population and comprises 90% of all pediatric age renal tumors [42]. This tumor has a marked propensity for macrovascular invasion which is detected in 35% of the patients with an IVC extension up to 10% [2, 42]. The tumor thrombus is classified based on its extent in the vessel system, and treatment approaches may vary based on this extension [42]. Most patients with venous invasion are asymptomatic at the time of the diagnosis, and the detection of endovenous tumor thrombus is made on initial imaging workup. Both CT and MRI may accurately detect the burden and the extent of the tumor thrombus. Both CT and MRI were shown to provide additional information to sonographic exam in local staging [43]. The preoperative detection of cavoatrial thrombus is important for decision-making process for clinical management [42].

**Fig. 12:**
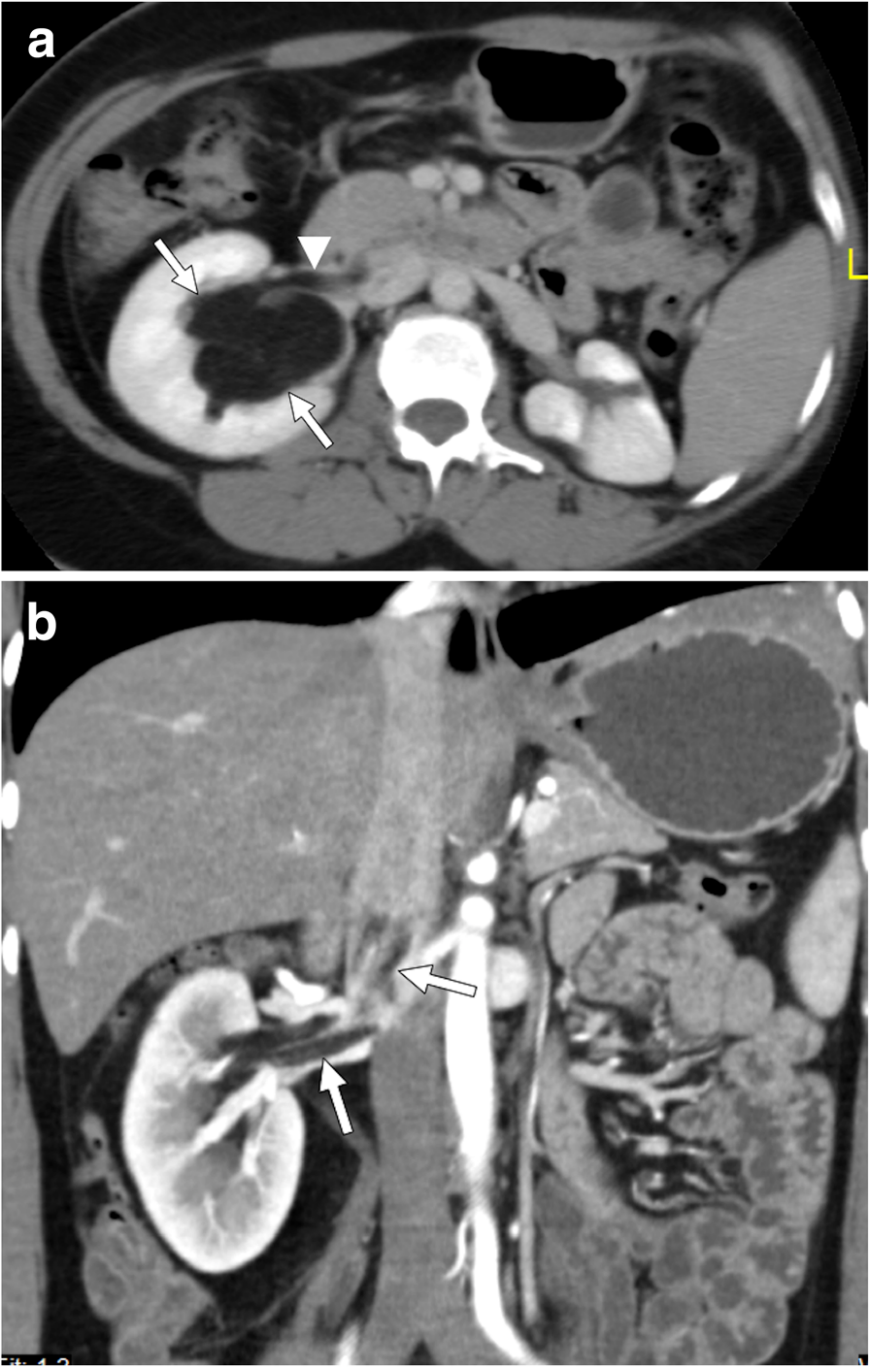
A 43-year-old female with no past medical history presented with right flank pain, shortness of breath, and microscopic hematuria. **a** Axial plane postcontrast CT image demonstrates expansile macroscopic fat containing mass (arrows) located within the right renal sinus suggestive for AML. Also, note is made of tumor thrombus extending into the right renal vein (arrowhead). **b** Coronally reformatted postcontrast CT image better delineates the extension of tumor thrombus into the right renal vein and IVC (arrows). Surgery confirmed the imaging findings

Renal angiomyolipomas (AML) are benign mesenchymal neoplasms originating from perivascular epithelioid cells with variable amounts of adipose tissue, smooth muscle tissue, and dystrophic vessels. The tumors are sporadic in great majority of the patients, and middle-aged women are the most commonly affected population [44]. Renal vein invasion by AMLs is a rare but known complication of these tumors. The presence of this venous invasion does not automatically indicate malignant nature of the tumor [44]. The large size and central location are known predisposing factors for renal vein invasion (Fig. 12). The frequent occurrence of this rare phenomenon on the right side may relate to the shorter and straighter course of the right renal vein [45]. The fat containing intravascular tumor may embolize to the lungs via the pulmonary arteries, but this complication is exceptionally rare with only few reported cases [46, 47].

Only a small portion (5%) of leiomyosarcomas arise from the blood vessels with the vast majority of them originate from IVC [48]. The disease is most commonly detected in women in their sixth to seventh decades [48, 49]. Renal vein leiomyosarcoma is even a rarer subgroup of a very rare disease, and due to this rarity, there is no information in the medical literature about its imaging and clinical characteristics. It may be frequently diagnosed as a tumor embolus from a primary RCC owing to its much higher frequency. The size may be an important imaging clue as the RCC dimension is greater than 7 cm in the majority of the patients (88%) with renal vein invasion [36]. In contrast to tumor thrombi due to RCCs, renal vein leiomyosarcomas have an average of 5.5 cm in size, and the intravascular component of the tumor was reported to be larger than its extravascular counterpart [48]. This rare tumor is more commonly on the left side, mostly related to longer length of this vessel, and the enhancement pattern is mostly in a homogenous fashion, but it is impossible to generalize this feature to all patients due to rarity of the disease [48].

**Fig. 13:**
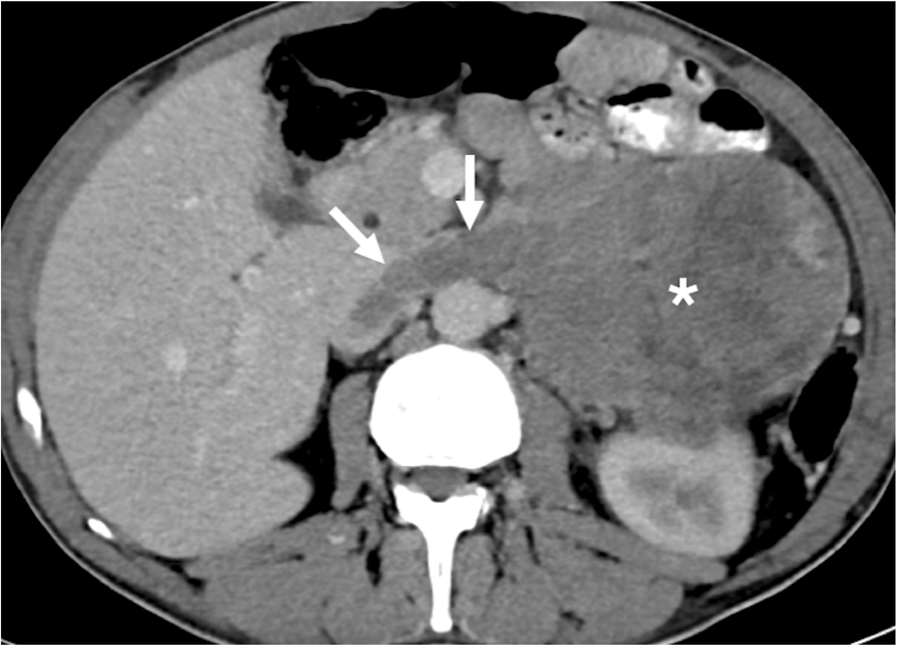
A 48-year-old male with recent onset HT and palpable abdominal mass. Axial plane postcontrast CT image shows a heterogeneously enhancing solid mass in the left adrenal fossa (asterisk). There was also extension of the tumor into the left renal vein (arrows) and IVC. Postsurgical histopathologic evaluation confirmed adrenocortical carcinoma with tumor thrombus in the left renal vein

The adrenal gland is a common site for metastatic disease for primary lung cancers. Tumor thrombus in renal veins is a rare phenomenon, with only anecdotal case reports on the subject, in the literature [50]. The most plausible explanation is the extension of the tumor thrombus into the renal vein through the adrenal vein [50]. The tumor thrombus appears as a filling defect within these vessels extending from the adrenal gland into the renal vein (Fig. 13). The continuity of the tumor with the tumor thrombus and expansion of the involved veins, in contrast to bland thrombus, are key imaging features for the correct diagnosis.

### Gonadal veins

The gonadal veins originate from the venous plexus of the ovaries and the testes and course superiorly, along the anterior aspect of the psoas muscles and the ureters, to join the left renal vein, on the left, and IVC, on the right. They are small caliber vessels and may be easily unnoticed during a routine scan, and endoluminal pathologies may be easily missed. Despite the fact that endoluminal tumor thrombus is a rare occurrence, there are some rare circumstances where this condition is detected within the lumen of these vessels.

**Fig. 14:**
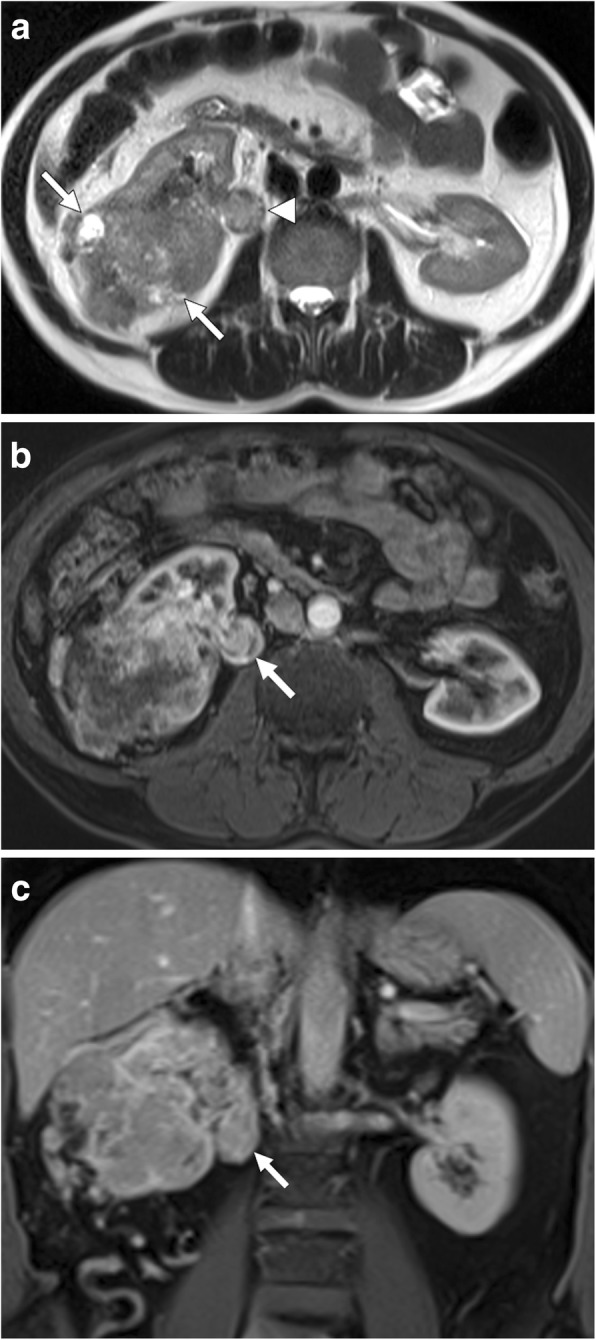
A 67-year-old male with no past medical history presented with right flank pain and hematuria. US study showed a large infiltrative mass filling right perirenal space. **a** Axial plane T2W MR image demonstrates a solid mass with heterogeneous signal intensity infiltrating the right kidney (arrows). Also, note is made of the expanded right testicular vein filled with thrombus (arrowhead) having a similar signal texture with the primary mass concerning for a tumoral thrombus over a bland thrombus. Axial (**b**) and coronal (**c**) plane postcontrast T1W MR images show enhancement within the thrombus (arrow) confirming its tumoral nature. Histopathological examination after surgical resection revealed primary RCC and tumoral thrombus within the testicular vein

RCC is very well known for its propensity to cause tumor thrombus within the IVC, but extension of RCC into the gonadal veins is rare. As the left gonadal vein drains into the left renal vein, endovascular tumor in the renal vein may retrogradely advance into the left gonadal vein [51]. The diagnosis may be difficult, and special attention should be paid to detect the tumor thrombus in axial and coronal images. Intraluminal tumor thrombus in the gonadal vein presents on contrast-enhanced CT or MRI as a tubular shaped filling defect within the vessel lumen, located anterior to the psoas muscle (Fig. 14).

**Fig. 15:**
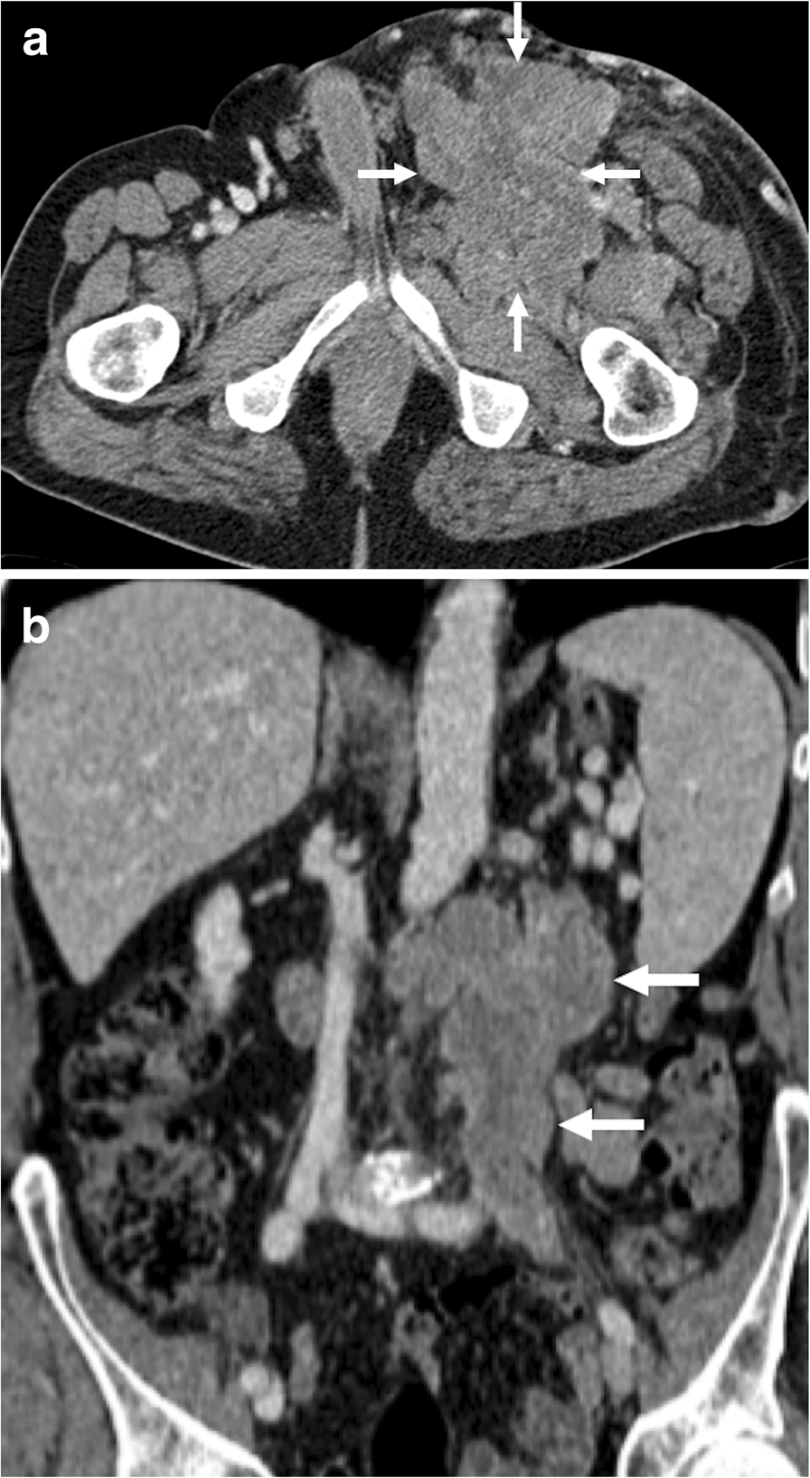
A 64-year-old male with no past medical history presented with left groin pain and palpable mass. **a** Axial plane postcontrast CT image demonstrates a large, multilobulated solid mass (arrows) in the left groin. **b** Coronally reformatted postcontrast CT image from the same study showed extension of this mass into the abdomen along the trajectory of the gonadal vessels (arrows). The patient underwent surgical resection, and histopathologic examination revealed leiomyosarcoma of the testicular vein. The left testicular artery could be seen separately from the primary mass

Gonadal vein leiomyosarcomas are exceedingly rare with around ten cases reported in the literature [52]. These tumors generally present as large masses in the retroperitoneum along the course in a longitudinal fashion and replace the gonadal vein. The tumor generally enhances strongly with internal necrotic areas. The tumor extension into the left renal vein may be easily detected with reformatted images in different planes [53]. To the best of our knowledge, a testicular vein leiomyosarcoma has not been reported before, and our case may be the first presented case (Fig. 15).

### Pelvic veins

Uterine tumors rarely give rise to venous tumor thrombus [54]. However, the presence of venous tumor thrombus is an important prognostic indicator, and surgical treatment becomes more comprehensive and complex compared to standard tumor surgery. The detection and evaluating the level of extension is critical for surgical planning.

**Fig. 16:**
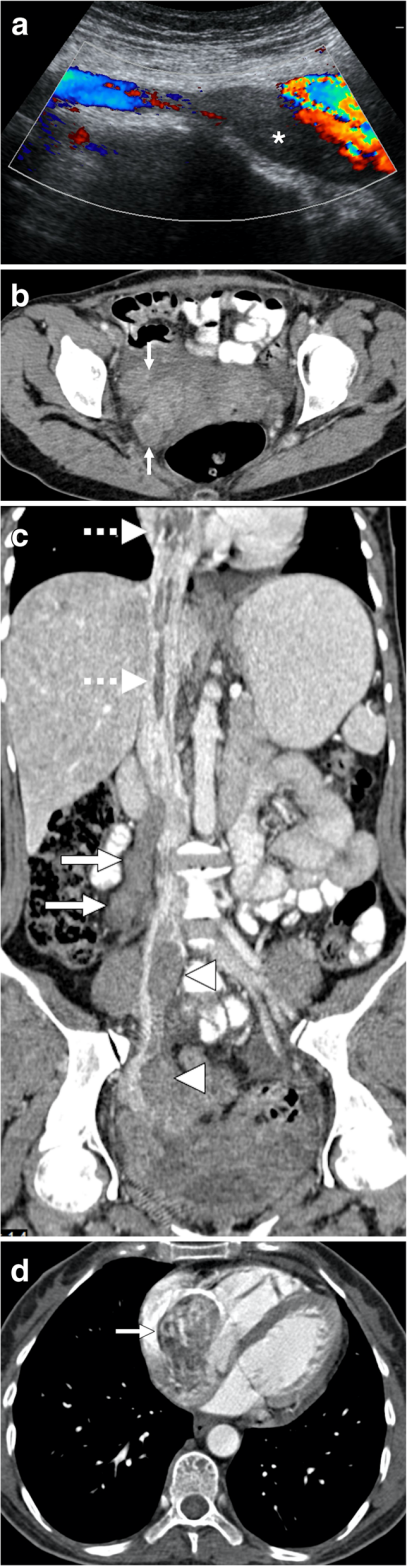
A 33-year-old female who underwent laparoscopic myomectomy 2 months ago now presents with right lower extremity edema, shortness of breath, and mild abdominal pain. **a** Color Doppler US image demonstrates thrombus within the lumen of the right common iliac vein (asterisk). **b** Axial plane postcontrast CT image shows tubular structures (arrows) in the pelvis suggestive for thrombosed adnexal veins. **c** Coronally reformatted postcontrast CT image of the same patient demonstrates thrombus in the right gonadal vein (arrows) and right common iliac vein (arrowheads). Thrombus was also noted in the hepatic and suprahepatic segments of the IVC (dotted arrows) with extension into the right atrium. **d** The tumor thrombus within the right atrium (arrow) is better appreciated on axial postcontrast image. Findings were found to be suggestive for intravascular leiomyomatosis, which was confirmed after histopathological exam of the pathologic specimen

Intravascular leiomyomatosis (IVL) is a rare tumor with around 200 cases reported since its first original description in 1896 [55]. Histopathologically, the tumor is composed of benign smooth muscle proliferation with an unusual pattern of growth, and the biological behavior of the tumor is aggressive [56]. The tumor is located in the pelvic or systemic veins. IVL is thought to arise from either from a benign uterine leiomyoma and, less commonly, de novo within the pelvic veins as a result of intimal smooth muscle proliferation [55]. Contiguous growth of the tumor may extend into the uterine vein, internal iliac veins, and IVC. Benign metastasizing leiomyomatosis (mostly to the lung) and disseminated peritoneal leiomyomatosis may be associated with IVL following surgery [57]. The detection of contrast enhancement within the thrombus is the key distinguishing feature of the IVL from bland caval thrombus (Fig. 16). Other sources of endovenous tumor thrombi, such as RCC, ACC, and Wilms tumor, must also be checked before the final diagnosis. The tumor may even extend into the heart to involve the right atrium (45.6% of the cases), right ventricle (45.6%), or the pulmonary vasculature (8.8%) [58].

**Fig. 17:**
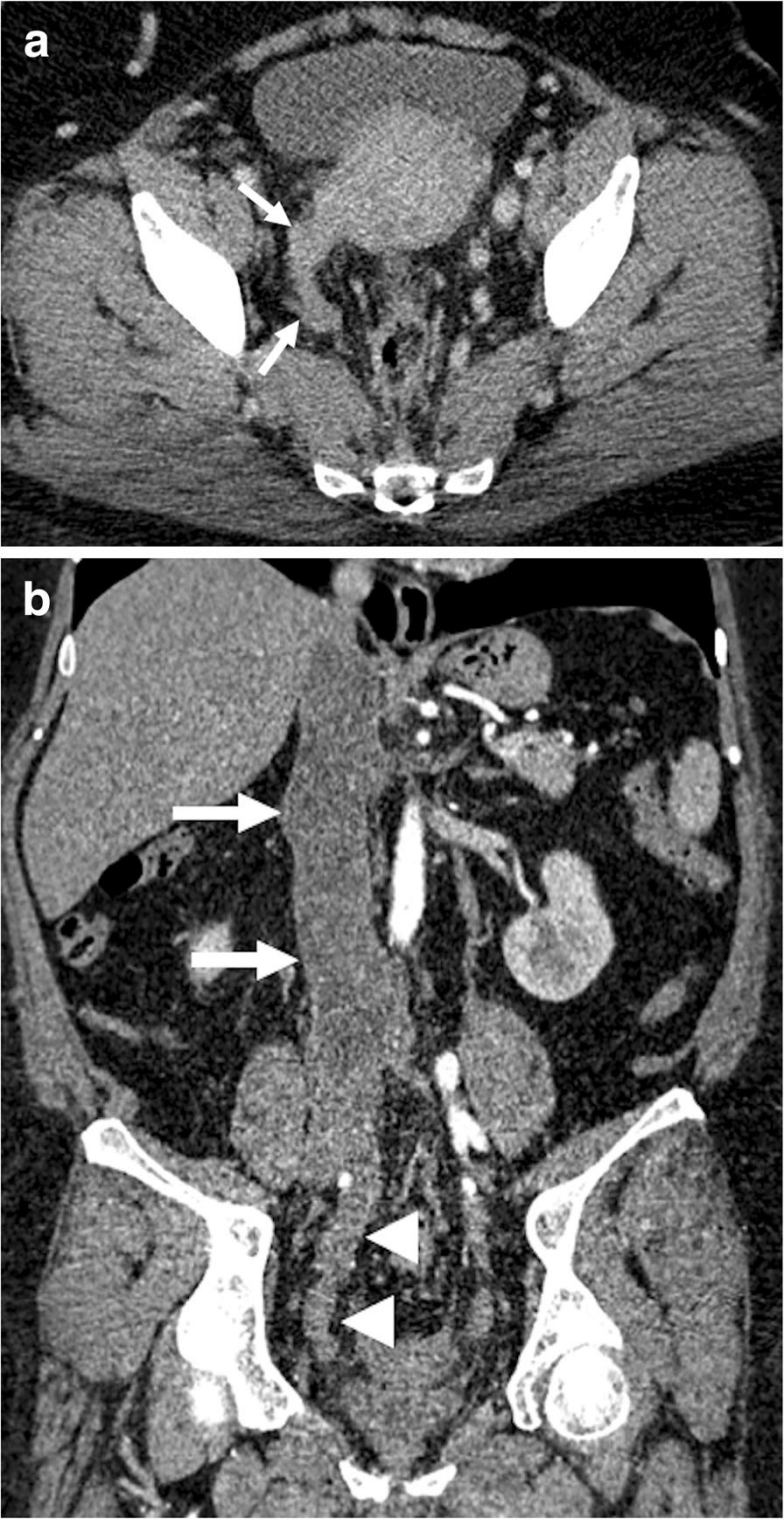
A 60-year-old female with no past medical history presented with lower extremity edema and postmenopausal bleeding. Endometrial sampling confirmed ESS. **a** Axial plane postcontrast CT image demonstrates the enlarged uterus with heterogeneous enhancement. Also, noted was thrombus in the right adnexal vein (arrows) with similar density of the heterogeneously enhancing uterus. **b** Coronally reformatted postcontrast CT image demonstrates extension of this thrombus along the right internal iliac vein (arrowheads) and IVC (arrows). Histopathologic examination after surgery confirmed the tumoral nature of the internal iliac and caval thrombus

Endometrial stromal sarcoma (ESS) is a relatively rare tumor constituting 0.2 to 1% of all uterine malignancies with an annual reported incidence of approximately 2 in 1,000,000 women [59]. Due to similar imaging features, ESS cases may be misdiagnosed with, far more common, uterine fibroids, and therapeutic delays are not uncommon [60]. Extrauterine extension of the ESS is not rare, with worm-like plugs within the vessels, but obvious intravascular extension of ESS is a rare clinical event with around 20 cases reported [54]. The endovenous tumor extension may be asymptomatic, but pulmonary embolism, and subsequent sudden cardiac death, is an important clinical concern in these patients. Both CT and MRI may be helpful for detecting and mapping the endovenous tumor thrombus (Fig. 17). Differentiation from bland thrombus may be challenging in some cases. CT may be especially useful for allowing to view the tumor thrombus in several different reformatted images. The presence of contrast enhancement within the tumor thrombus appears to be helpful for differentiating tumor thrombus from its bland counterpart. Intravascular leiomyomatosis should also be considered in the differential diagnosis in patients with no histopathological proof for ESS, and pathologic examination of the tumor thrombus may be necessary for definitive diagnosis.

### Other vessels

#### Tumor thrombus in the inferior mesenteric vein

**Fig. 18:**
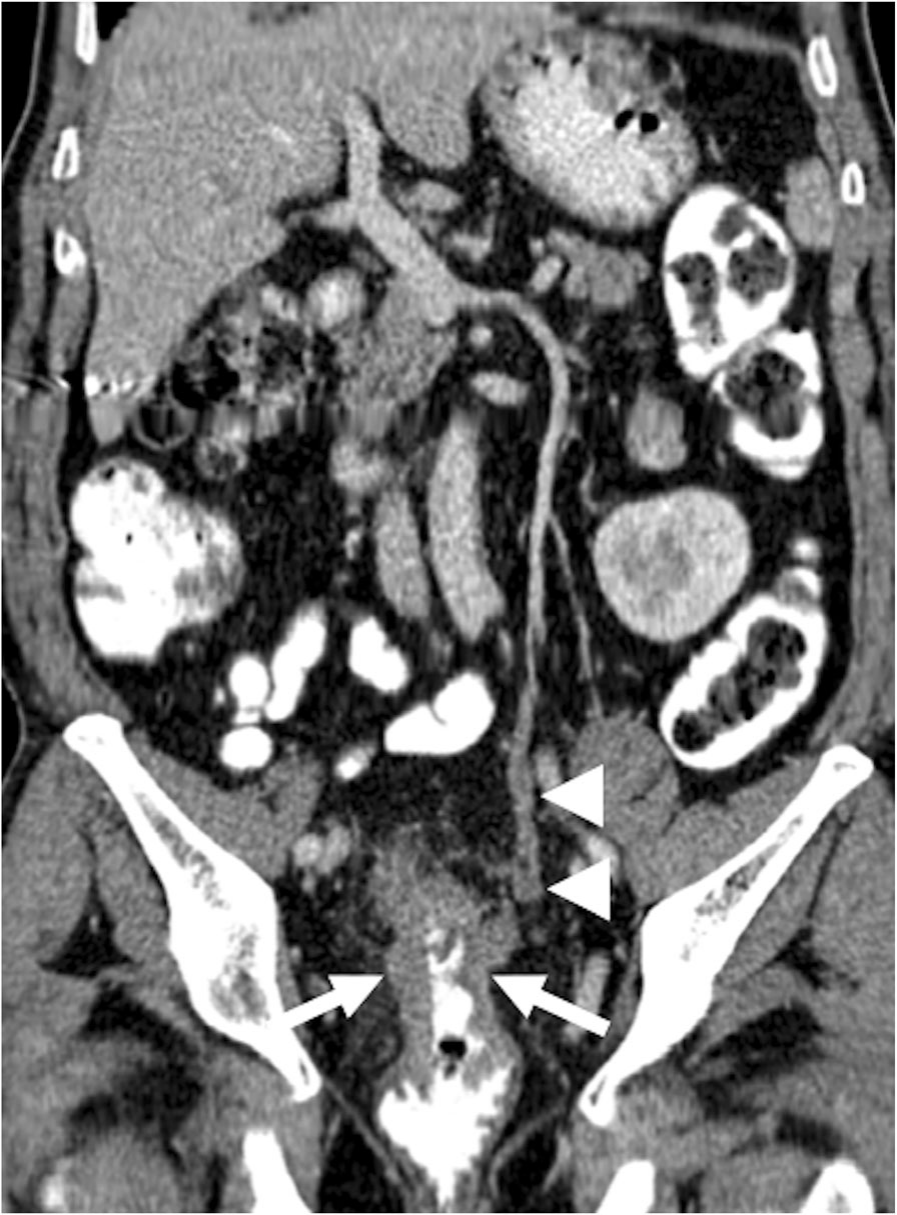
A 57-year-old male patient with no significant past medical history presented with abdominal pain and hematochezia. Colonoscopic examination and biopsy confirmed adenocarcinoma of the rectum. Coronally reformatted postcontrast CT image demonstrates the primary tumor (arrows) and thrombus within the lumen of the caudal portion of the IMV (arrowheads). Surgical resection and histopathologic examination confirmed the tumoral thrombus in the resected portion of the IMV

In patient with colorectal cancer, venous tumor thrombosis formation is a rare and unusual route for tumor dissemination [61]. It was speculated that the presence of venous tumor thrombus due to colorectal cancer carries a higher risk for liver metastases [62]. CT with its unique multiplanar reformatting skills appears to be most well-suited imaging modality for detecting this rare phenomenon (Fig. 18).

#### Leiomyosarcoma of the great saphenous vein

**Fig. 19:**
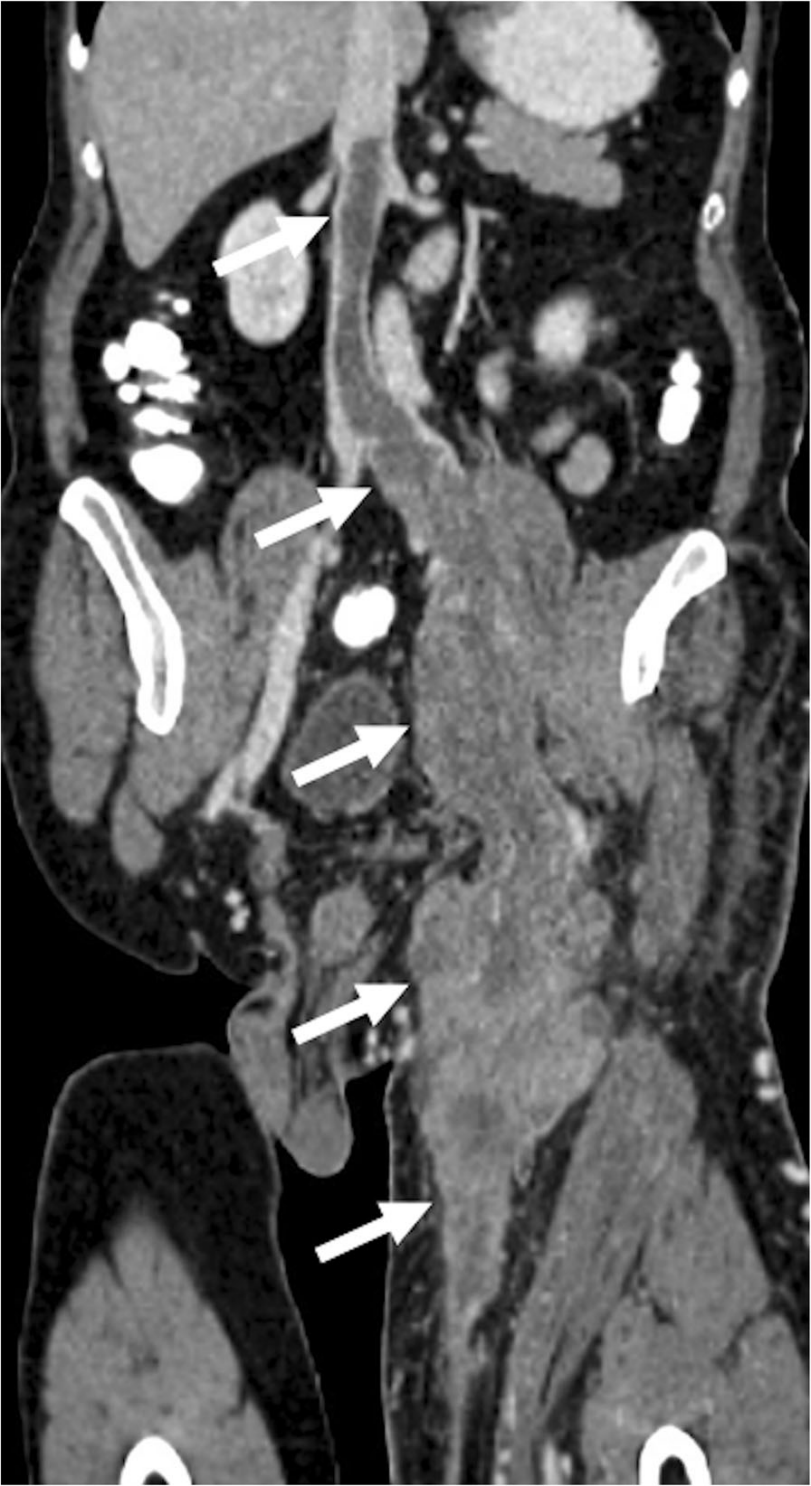
A 65-year-old male with no significant past medical history presented with left lower extremity swelling and palpable groin mass. Coronally reformatted postcontrast CT image demonstrates heterogeneously enhancing solid mass extending along the left great saphenous vein. The mass extends cranially to the IVC via the femoral and iliac veins (arrows). Postsurgical histopathologic examination revealed leiomyosarcoma of the great saphenous vein

Leiomyosarcoma of the great saphenous vein is an extremely rare tumor with around less than 50 cases since its first original description 1868 by Aufrecht [63]. Due to its rarity, there is no statistical information about these extremely rare tumors, and most data are derived from anecdotal case reports. The majority of these tumors are located above the knee, and below the tumors are exceedingly rare [63]. Both CT and MRI may be useful for detection the local disease as well as distant metastatic foci [64, 65] (Fig. 19). MRI may be more useful to evaluate the soft tissue planes of the extremities [64, 66]. CT and MR angiography may also be used for diagnosis and evaluating the local extension of the disease.

## Conclusion

Tumor thrombus within the abdominal veins may be easily detected with modern imaging techniques. Both MRI and CT angiographic studies may be extremely helpful both to detect and to evaluate the extension of the disease. As this is a rare clinical situation, excluding tumor thrombi due to HCC and RCC, they may be easily overlooked or misdiagnosed. The early and accurate detection of endovenous tumor thrombi significantly alter the surgical approach and prognostic stratification and, therefore, they should be accurately reported on imaging studies.

## Data Availability

Data sharing is not applicable to this article as no datasets were generated or analyzed during the current study.

## Abbreviations

ACC: Adrenocortical carcinoma
AML: Angiomyolipomas
CT: Computed tomography
DWI: Diffusion-weighted imaging
ESS: Endometrial stromal sarcoma
FDG-PET: Fluorodeoxyglucose positron emission tomography
HCC: Hepatocellular carcinoma
IMV: Inferior mesenteric vein
IVC: Inferior vena cava
IVL: Intravascular leiomyomatosis
MRI: Magnetic resonance imaging
PNET: Pancreatic neuroendocrine tumors
PV: Portal vein
RCC: Renal cell carcinoma
T1W: T1-weighted
T2W: T2-weighted
